# Shikimic Acid Production in *Escherichia coli*: From Classical Metabolic Engineering Strategies to Omics Applied to Improve Its Production

**DOI:** 10.3389/fbioe.2015.00145

**Published:** 2015-09-23

**Authors:** Juan Andrés Martínez, Francisco Bolívar, Adelfo Escalante

**Affiliations:** ^1^Departamento de Ingeniería Celular y Biocatálisis, Instituto de Biotecnología, Universidad Nacional Autónoma de México, Cuernavaca, Mexico

**Keywords:** *Escherichia coli*, metabolic engineering, shikimic acid, transcriptome, metabolome, antiviral drug, influenza

## Abstract

Shikimic acid (SA) is an intermediate of the SA pathway that is present in bacteria and plants. SA has gained great interest because it is a precursor in the synthesis of the drug oseltamivir phosphate (OSF), an efficient inhibitor of the neuraminidase enzyme of diverse seasonal influenza viruses, the avian influenza virus H5N1, and the human influenza virus H1N1. For the purposes of OSF production, SA is extracted from the pods of Chinese star anise plants (*Illicium* spp.), yielding up to 17% of SA (dry basis content). The high demand for OSF necessary to manage a major influenza outbreak is not adequately met by industrial production using SA from plants sources. As the SA pathway is present in the model bacteria *Escherichia coli*, several “intuitive” metabolically engineered strains have been applied for its successful overproduction by biotechnological processes, resulting in strains producing up to 71 g/L of SA, with high conversion yields of up to 0.42 (mol SA/mol Glc), in both batch and fed-batch cultures using complex fermentation broths, including glucose as a carbon source and yeast extract. Global transcriptomic analyses have been performed in SA-producing strains, resulting in the identification of possible key target genes for the design of a rational strain improvement strategy. Because possible target genes are involved in the transport, catabolism, and interconversion of different carbon sources and metabolic intermediates outside the central carbon metabolism and SA pathways, as genes involved in diverse cellular stress responses, the development of rational cellular strain improvement strategies based on omics data constitutes a challenging task to improve SA production in currently overproducing engineered strains. In this review, we discuss the main metabolic engineering strategies that have been applied for the development of efficient SA-producing strains, as the perspective of omics analysis has focused on further strain improvement for the production of this valuable aromatic intermediate.

## Introduction

**Figure 1:**
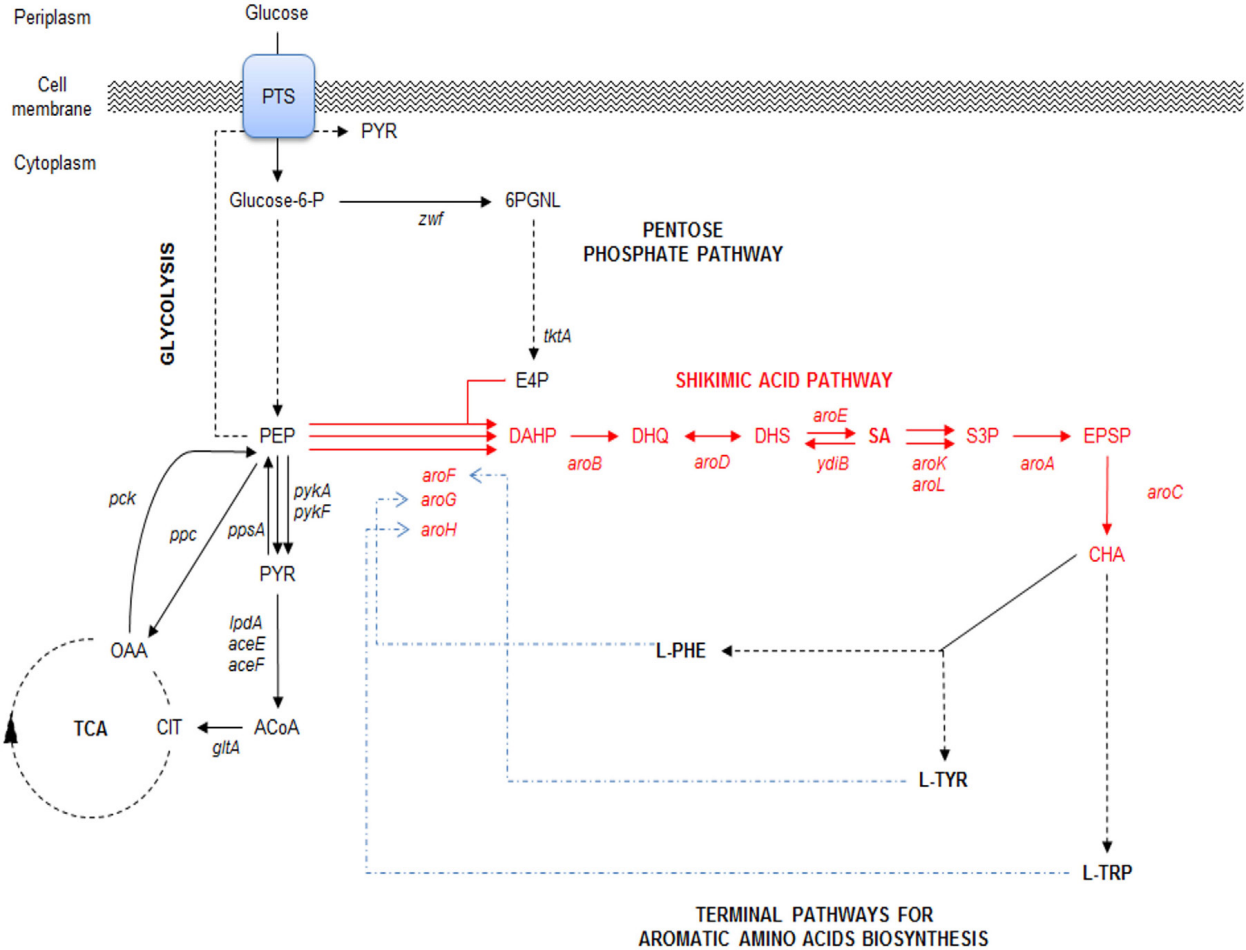
**Schematic representation of the main glucose transport system, central carbon metabolism (CCM) (glycolysis and pentose phosphate pathways), their interconnection with SA pathway and final aromatic amino acids pathway in *E. coli***. PTS, phosphotransferase:PEP:glucose system. CCM key intermediates and protein encoding genes: TCA, tricarboxylic acid pathway; E4P, erythrose-4-P; PGNL, 6-phospho d-glucono-1,5-lactone; PEP, phosphoenolpyruvate; PYR, pyruvate; ACoA, acetyl-CoA; CIT, citrate; OAA, oxaloacetate; *zwf*, glucose 6-phosphate-1-dehydrogenase; *tktA*, transketolase I; *pykA*, *pykF*, pyruvate kinase II and pyruvate kinase I, respectively; *lpdA*, *aceE*, and *aceF*, coding for PYR dehydrogenase subunits; *gltA*, citrate synthase; *pck*, PEP carboxykinase; *ppc* PEP carboxylase; *ppsA*, PEP synthetase. SA pathway intermediates and genes: DAHP, 3-deoxy-d-*arabino*-heptulosonate-7-phosphate; DHQ, 3-dehydroquinate; DHS, 3-dehydroshikimate; SA, shikimic acid; S3, SHK-3-phosphate; EPSP, 5-enolpyruvyl-shikimate 3-phosphate; CHA, chorismate; *aroF*, *aroG*, *aroH*, DAHP synthase AroF, AroG and AroH, respectively; *aroB*, DHQ synthase; *aroD*, DHQ dehydratase; *aroE* and *ydiB*, SHK dehydrogenase and SHK dehydrogenase/quinate dehydrogenase, respectively; *aroA*, 3-phosphoshikimate-1-carboxyvinyltransferase; *aroC*, CHA synthase. Terminal aromatic amino acids products: l-TRP, l-tryptophan; l-PHE, l-phenylalanine; l-TYR, l-tyrosine. Continuous arrows indicate single enzymatic reactions; dashed arrows show several enzymatic reactions; dashed-dotted arrows (blue) show repression of DAHPS isoenzymes allosteric regulatory circuits. Adapted from Keseler et al. (2013) and Rodriguez et al. (2014).

**Figure 2:**
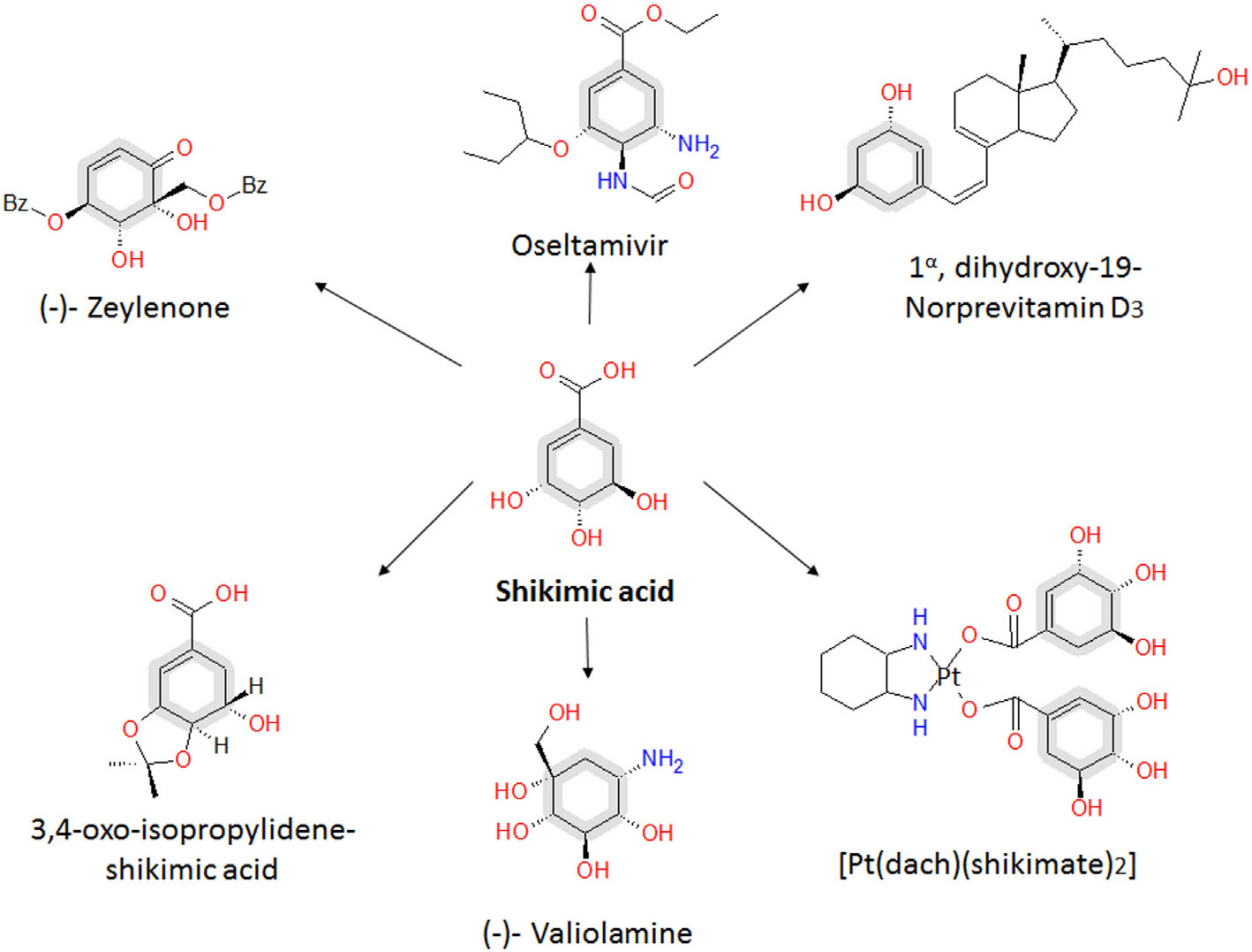
**Relevant SA derivatives with high added value**. OSF, viral inhibitor of diverse influenza virus types, including seasonal types A and B, avian virus H5N1, and human virus H1N1. (−)-Zeylenone is a compound with antiviral, anticancer, and antibiotic activities. (−)-Valiolamine, a very strong α-glucosidase with inhibitory activity against porcine intestinal enzymes sucrase, maltase, and isomaltase. [PT(datch)(SA)_2_] is an active compound against L1210 leukemia. 3,4-Oxo-isopropylidene-SA, with antithrombotic activity and anti-inflammatory effects. Analogs of 1α, dihydroxy-19-Nor previtamin D3 is a compound with promising applications in the treatment of osteoporosis and malignancies. Adapted from Estevez and Estevez (2012) and Diaz Quiroz et al. (2014).

Compounds derived from the aromatic amino acid (AA) pathway play important roles in the pharmaceutical and food industries as raw materials, additives, or final products (Patnaik et al., 1995; Bongaerts, 2001; Báez et al., 2001; Yi et al., 2002; Chandran et al., 2003; Báez-Viveros et al., 2004; Gosset, 2009). This metabolic pathway is present in bacteria and plants, starting with condensation of the central carbon metabolism (CCM) intermediates phosphoenolpyruvate (PEP) and erythrose-4-phosphate (E4P) to form the first AA pathway intermediate d-*arabino*heptulosonate-7-phospate (DAHP). From this compound to chorismic acid (CHA), the pathway is mostly linear and represents the first part of the AA pathway, known as the common AA pathway or the shikimic acid (SA) pathway (Figure 1). One of the specific intermediates on this pathway is SA, which is a highly functionalized six-carbon cyclic compound with three asymmetric centers. Therefore, SA is an enantiomeric precursor for the production of many high valuable biological active compounds for different industries. SA is the precursor for the synthesis of compounds with diverse pharmaceutical applications, including as an antipyretic, antioxidant, anticoagulant, antithrombotic, anti-inflammatory, or analgesic agent, for the synthesis of anticancer drugs, such as (+)-zeylenone (which has been shown to inhibit nucleoside transport in Ehrlich carcinoma cells and to be cytotoxic to cultured cancer cells), and for antibacterial or hormonal applications [reviewed in Estevez and Estevez (2012), Liu et al. (2012), and Diaz Quiroz et al. (2014)] (Figure 2).

Specifically, SA has great pharmaceutical relevance because it is the precursor for the chemical synthesis of oseltamivir phosphate (OSF), known as Tamiflu^®^, used as the antiviral inhibitor of the neuraminidase enzyme for the treatment of diverse seasonal influenza viruses, including influenza A and B, the avian influenza virus H5N1, and the human influenza virus H1N1 (Krämer et al., 2003; Estevez and Estevez, 2012; Ghosh et al., 2012; Diaz Quiroz et al., 2014). For this purpose, SA is obtained from the seed of the Chinese star anise plant *Illicium verum*, which contains between 2 and 7% of the intermediate. However, it can only be retrieved from plants after 6 years of crop growth and harvested in September and October (Li et al., 2007; Raghavendra et al., 2009; Wang et al., 2011). To recover SA from the seed, a 10-step process is required, taking ~30 kg of seed to produce 1 kg of SA. According to Li et al. (2007) on their 2007 patent, ~90% of the Chinese harvest is used by Roche (2009) for OSF production.

In 2009, Roche reported Tamiflu^®^ sales to be 3.5 billion dollars, with a production capacity of up to 33 million treatments per month and 400 million packages per year (Scheiwiller and Hirschi, 2010). For the antiviral production, up to 1.3 g of SA are required to manufacture 10 doses to treat only one person, estimating a production requirement for this antiviral drug alone of ~520,000 kg/year (Rangachari et al., 2013). Even so, this reported production capacity could be insufficient in the case of an influenza pandemic, particularly with more pathogenic and infective strains. An estimated production of 30 billion doses, requiring 3.9 million kilograms of SA, would be necessary to cover a severe influenza outbreak (Rangachari et al., 2013). According to the World Health Organization regarding influenza outbreak preparedness, only 66 million people in medium to low income countries are covered up with antiviral stocks, representing only 2.25% of the populations in these countries (World Health Organization, 2011). This situation results in a possibly low production capacity since in 2010, 100 million people were infected with common strains of influenza in Europe, Japan, and the United States alone. Moreover, before 2010, pandemic influenza has affected between 20 and 40% of the population, causing over 20 million deaths (Scheiwiller and Hirschi, 2010; World Health Organization, 2011).

For the reasons mentioned before and due to the relevance of SA in diverse industrial setups, many studies concerning SA production have been conducted within the past years, resulting in new and insightful strategies for its production, including recovery technologies, chemical synthesis methods, and biotechnological production methods using microorganisms. In fact, one of the most studied alternatives for SA production processes is biotechnological synthesis using recombinant microbial strains that are capable of producing high yields and that have high productivities, as there are key advantages over chemical synthesis, which include environmental friendliness, the availability and abundance of low-cost renewable feed stocks, and selectivity and diversity of the obtained products (Chen et al., 2013). These strains can be obtained by genetic modification, altering cellular properties to enhance their production capacity through the application of diverse metabolic engineering (ME) approaches (Krämer et al., 2003; Ghosh et al., 2012; Diaz Quiroz et al., 2014). However, despite the great achievements accomplished through this discipline, performance improvement has become limited after the first breakthroughs, mainly because of the traditional local pathway modification strategies. This is probably due to the limited understanding of the overall mechanism of metabolic regulation (Matsuoka and Shimizu, 2012). Therefore, given the importance of finding not only a particular pathway but also global information regarding cell physiology and metabolism to overcome production limitations, a systems biology approach supported by omics data may be the solution for improving SA production. The goal of this work is not only to review the literature on the great biotechnological achievements made for SA production, mainly in *Escherichia coli*, but also to outline future perspectives on research performed in the omics era, which could provide relevant tools for understanding cell behavior and production optimization via biotechnological processes.

### Classical metabolic engineering approaches for SA production

Metabolic engineering has been used since 1991 for strain modification by using recombinant DNA technology to enhance the production of specific metabolites (Matsuoka and Shimizu, 2012). The efforts to use ME have extended from the early years to optimize many cellular behaviors or parameters, such as substrate consumption, robustness, and tolerance toward toxic compounds and media conditions (Matsuoka and Shimizu, 2012). Classical ME strategies for strain development include various steps, such as the selection of a proper organism, elimination of competing pathways, deregulation of desired pathways at the enzyme activity and transcriptional levels, and overexpression of enzymes at flux bottlenecks (Patnaik et al., 1995). Regarding the selection of an organism, *E. coli* has been preferred for industrial purposes and ME applications because of the knowledge available on *E. coli* physiology and the great numbers of tools developed to modify its genome (Chen et al., 2013). Therefore, many advances had been made regarding SA in *E. coli*, rendering strains capable of being used in industrial applications (Frost et al., 2002; Li et al., 2007).

In *E. coli*, the SA pathway starts by condensation of the CCM intermediates PEP and E4P by three DAHP synthase isoenzymes, AroG, AroF, and AroH (coded by *aroG*, *aroF*, and *aroH*, respectively), to produce DAHP. These three isoenzymes are responsible for the redirection from CCM intermediates toward the synthesis of aromatic compounds and are allosterically regulated specifically by the final products of AA biosynthesis. AroG catalyzes ~80% of DAHPS activity and is specifically feedback regulated by l-phenylalanine, AroF (~20% of DAHPS activity) is feedback regulated by l-tyrosine, and AroH (~1% of DAHPS activity) is regulated by l-tryptophan. Additionally, the transcription of *aroG* and *aroF* is controlled by the *tyrR* repressor, with the end products of the AA pathway (l-phenylalanine and l-tyrosine, respectively) acting as corepressors, whereas the transcription of *aroH* is controlled by the *trpP* repressor, with l-tryptophan acting as a corepressor (Keseler et al., 2013) (Figure 1). The ME solution for this first flux bottleneck is the expression of a DAHP AroG and AroF synthase that is not sensitive to feedback inhibition (fbr) (AroG^fbr^ and AroF^fbr^). Mutations in the *aroG* and *aroF* genes lead to l-phenylalanine and l-tyrosine feedback-insensitive mutants with increased net carbon flux from CCM to the SA pathway (Keseler et al., 2013; Lin et al., 2014; Rodriguez et al., 2014); these mutants have been used in most SA production strains (Table 1) (Chandran et al., 2003; Escalante et al., 2010; Chen et al., 2012, 2014; Rodriguez et al., 2013). Forward reactions convert DAHP to dehydroquinic acid (DHQ), then to 3-dehidroquinate (DHS) and finally to SA by the enzymes 3-dehydroquinate synthase (*aroB*), 3-dehydroquinate dehydratase (*aroD*), and shikimate dehydrogenase (*aroE*), respectively (Figure 1). Although the pathway to SA conversion is small and linear, its regulation and the competition for precursor metabolites remain quite complicated because the SA pathway is dependent on the glycolytic and pentose phosphate pathways (PPPs) to provide the starting precursors PEP and E4P, respectively (Gosset, 2009; Escalante et al., 2012; Ghosh et al., 2012; Rodriguez et al., 2014).

**Table 1:** **Relevant *E. coli* engineered strains for SA production**.

Producing strain	Phenotypic traits	Special comments	Culture conditions	Title (g/L)	Yield (mol SA/mol glc)	Reference
SA112	BW25113Δ*aroKL*, P*_pps_*::P*_lac_*Q1,P*_csrB_*::P*_lac_*Q1Pt5-*pps*,P*_T5_*-*csrB*, 5P*_tac_*-*tktA*	CIChE evolved to optimize SA production	Shake flasks cultures with 10 g/L glc, 1 g/L peptone	1.70	0.25	Cui et al. (2014)
DHPYAAS-T7	DH5α Δ*ptsHIcrr*, Δ*aroKL*, Δ*ydiB* pAOC-TGEFB:*aroE*, *aroB*, *glk*, *tkta*, *aroF*^fbr^	Plasmid overexpression of SA related genes	Shake flasks cultures (50 mL), M9 broth supplemented with 25 g/L glycerol, 10 g/L peptone, 15 g/L YE	1.066	0.23	Chen et al. (2012)
PB12.SA22	JM101 Δ*ptsH*, *ptsI*, *crr*::Km^r^ Δ*arokL*::cm^r^ pJLB*aroG*^fbr^*tktA*, pTOPO-*aroBaroE*	Laboratory evolved PTS^−^ glucose^−^ into glucose^+^ derivative phenotype	1 L batch bioreactor 25 g/L glc and 15 g/L YE	7.05	0.22	Escalante et al. (2010)
SA5	B0013 Δ*arokL*::dif Δ*ptsG*::*dif* Δ*ydiB*:*dif* Δ*ackA-pta*::*dif* pTH-*aroG*^fbr^-*ppsA-tktA*	Plasmid over expression of SA related genes	7 L fed-batch bioreactor, initial 15 g/L glc supplemented with AA and vitamins	14.6	0.3	Chen et al. (2014)
SA114	BW25113Δ*aroKL*, P*_pps_*::P*_lac_*Q1, P*_csrB_*::_P_*lac*Q1 P*_T5_*-*pps*,P*_T5_*-*csrB*, 5P*_tac_*-*tktA*, 5P*_tac_*-*pntAB*	CIChE evolved to optimize SA	Shake flasks, 10 g/L glc, 1 g/L peptone	2.99	0.31	Cui et al. (2014)
SP1.1pts-/pSC6.090B	*DH5*α Δ*ptsH-ptsI-crr* Δ*serA*::*aroB* Δ*aroL*::*Tn10* Δ *aroK*::*Cm^r^ P_tac_glf glk*, *aroF*^fbt^*tktA*, *P^tac^aroE*, *serA*	Heterologous *glk* and *glf* from *Z. mobilis* to restore glc transport and phosphorylation in PTS^−^ glc^−^ phenotype	10 L fed-batch bioreactor 55–170 mM Glc + 15 g/L YE	84	0.33	Chandran et al. (2003)
SA116	BW25113Δ*aroKL*, *P_pps_*::*P_lac_Q1*, *P_csrB_*::*P_lac_Q1 P_T5_-pps*, *P_T5_-csrB*, 5*P_tac_-tktA*, 5*P_tac_-nadK*	CIChE evolved to optimize SA	Shake flasks 10 g/L glc, 1 g/L peptone	3.12	0.33	Cui et al. (2014)
AR36	*JM101* Δ*ptsH*, *ptsI*, *crr*::*Km^r^* Δ*arokL*::*cm^r^* Δ*pykF* Δ*lacI pTrcAro6-aroB*, *tktA*, *aroG^fbr^*, *aroE*, *aroD zwf*	Constitutive strong over expression by synthetic operon on plasmid	1 L batch bioreactor 100 g/L glc + 15 g/L YE	41.8	0.42	Rodriguez et al. (2013)

*glc, glucose; YE, yeast extract*.

SA production starts in CCM, further away from glucose consumption. In *E. coli*, the majority of glucose transport occurs via the PEP:glucose phosphotransferase system (PTS), which uses a phosphate group from one molecule of PEP to simultaneously import and phosphorylate periplasmic glucose, resulting into 6-phosphate glucose (G6P) and pyruvate (PYR) (Figure 1). For these reasons, the application of ME strategies only on the SA pathway would not render a significantly optimized strain for SA production (Ghosh et al., 2012).

To optimize productivity and yields from a given carbon source, modification of the CCM pathways supplying the needed precursors and energy sources for product synthesis is required (Patnaik and Liao, 1994). For the E4P supply, the PPP is the responsible for its production. The overexpression of transketolase I (TktA, coded by *tktA*) and transaldolase (coded by *talA*), resulting in the preferential use of TktA to improve the E4P pool for the synthesis of DAHP (Flores et al., 1996; Draths et al., 1999; Frost et al., 2002; Chandran et al., 2003; Escalante et al., 2010; Rodriguez et al., 2013).

Regarding increasing the PEP pool, the first problem arises with the consumption of 50% of the PEP resulting from the catabolism of one molecule of glucose-6-P by PTS during the translocation and phosphorylation of one molecule of glucose. A rational approach is to reconvert PYR to PEP by overexpressing PEP synthase (coded by *pps*); this solution, along with the expression of a DAHPS^fbr^ (AroG^fbr^ or AroF^fbr^), leads to a 51% (mol/mol) yield of DHS and related SA pathway metabolites. This yield is in fact higher than the 43% (mol/mol) yield calculated from stoichiometric reactions, reflecting the effective redistribution of the PEP to PYR pool ratio and the ability of the strain to redirect this new imbalance into the SA pathway (Yi et al., 2002; Chandran et al., 2003; Krämer et al., 2003; Escalante et al., 2010; Rodriguez et al., 2013). Overexpression of the *pps* gene has been studied; the maximum yield of SA is not obtained under the maximum concentration of the enzyme. In fact, it has been found that expression of this enzyme over the optimized level would only reduce the yields of SA intermediates, probably due to energetic imbalances (Yi et al., 2002).

The maximum theoretical yield limitation can be changed by restructuring the metabolic network, providing the system with a new stoichiometric matrix. Therefore, a natural solution for the PEP pool was to eliminate the PTS system, which would not only modify the amount of PEP but also redistribute the stoichiometric matrix to raise the maximum theoretical yield to 86% (mol/mol) (Chandran et al., 2003; Krämer et al., 2003). The main problem with this solution is the resultant low level of glucose transport, which results in a strain with hampered growth (PTS^−^ phenotype) (Flores et al., 1996, 2007; Aguilar et al., 2012). Nevertheless, various strategies have been developed to revert this low glucose consumption and low growth phenotype. Using rational ME strategies, substitution of the PTS for another glucose transport system has been performed. Chandran et al. used heterologous expression of the *Zymomonas mobilis* (Glf) glucose transporter, a *glf*-encoded glucose facilitator and a *glk*-encoded glucose kinase (Glk), thereby allowing cells to consume glucose more efficiently without consuming PEP (Frost et al., 2002; Chandran et al., 2003; Krämer et al., 2003). Another strategy is to apply laboratory adaptive evolution onto a PTS^−^ strain. Flores et al. used a continuous culture with glucose as a single carbon source to select high glucose consumption-evolved derivative strains (PTS^−^ glc^+^ phenotype). Characterization of these mutants revealed overexpression of the *galP* and *glk* genes encoding galactose permease and glucokinase, respectively, allowing and improving glucose transport and phosphorylation capabilities and resulting in an increased specific growth rate and PEP availability (Flores et al., 1996, 2007; Aguilar et al., 2012).

**Figure 3:**
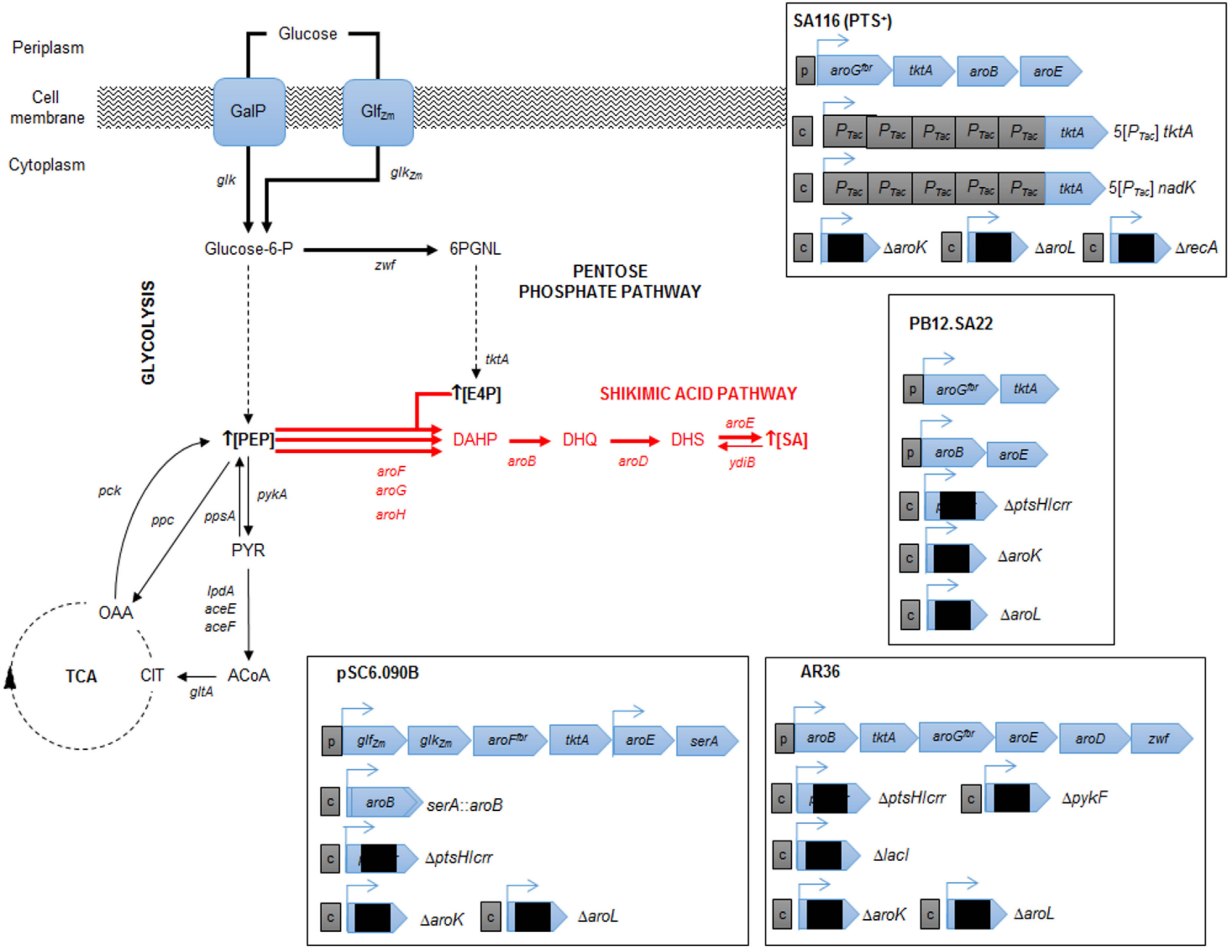
**Relevant engineered *E. coli* strains for SA production**. Metabolic traits of *E. coli* derivative strains engineered for SA production resulting in highest SA titer and yield from glucose. The figure illustrates alterative glucose transporter GalP (galactose permease) selected by the cell after laboratory adaptive evolution process of a PTS^−^ mutant (Flores et al., 1996, 2007; Aguilar et al., 2012). Glf (glucose facilitator) and Glk (glucokinase) from *Z. mobilis* (plasmid cloned). Resultant characteristics of engineered strains are shown for pSC6.090B (Chandran et al., 2003), PB12.SA22 (Escalante et al., 2010), AR36 (Rodriguez et al., 2013), and SA116 strain (Cui et al., 2014). CCM key intermediates and protein encoding genes: TCA, tricarboxylic acid pathway; E4P, erythrose-4-P; PGNL, 6-phospho d-glucono-1,5-lactone; PEP, phosphoenolpyruvate; PYR, pyruvate; ACoA, acetyl-CoA; CIT, citrate; OAA, oxaloacetate; *zwf*, glucose 6-phosphate-1-dehydrogenase; *tktA*, transketolase I; *pykA*, pyruvate kinase II; *lpdA*, *aceE* and *aceF*, coding for PYR dehydrogenase subunits; *gltA*, citrate synthase; *pck*, PEP carboxykinase; *ppc* PEP carboxylase; *ppsA*, PEP synthetase. SA pathway intermediates and genes: DAHP, 3-deoxy-d-*arabino*-heptulosonate-7-phosphate; DHQ, 3-dehydroquinate; DHS, 3-dehydroshikimate; SA, shikimic acid. Continuous arrows indicate single enzymatic reactions; dashed arrows show several enzymatic reactions. Bold arrows show improved carbon flux. Black squares in plasmids/operons indicate gene interruption; c, chromosomal gene interruption or integration; p, plasmid-cloned genes.

Finally, with precursors known to induce redirection, deregulation, and overexpression of the SA pathway genes a*roB*, *aroD*, and *aroE* have been achieved, resulting in an efficient carbon flux from CCM to the SA pathway. The highest production to date corresponds to the SP1.1pts-/pSC6.090B strain, a PTS^−^ derivative strain with a plasmid containing two *tac* promoters, the first of which controls expression of the *glf*, *glk*, *aroF*^fbr^, and *tktA* genes and the second of which controls expression of the *aroE* and *serA* genes (Chandran et al., 2003). The reasoning behind this construction was to increase the PEP pool by deleting PTS, to recuperate glucose consumption by overexpressing *glf* and *glk*, to assure E4P pool enhancement by overexpressing *tktA* and to induce a deregulated pull toward the AA pathway via an *aroF*^fbr^, as discussed before. The second promotor in the plasmid was designed to overexpress *aroE*, allowing continuous flux of the SA pathway; additionally, a second copy of *aroB* was introduced into the chromosome instead of reintroducing the serine production-related gene *serA* to the cell via the plasmid and was used as a selection marker for plasmid retention. This approximation, along with the deletion of genes related to SA consumption (*aroK* and *aroL*), allowed SA accumulation, achieving a production capacity of 87 g/L SA, with a yield of 36% (mol/mol) and a productivity of ~5.3 g/L h when a 10 L glucose-fed batch was cultured. This strain has the highest titer accumulation recorded in the literature to date (Table 1; Figure 3).

Even with these rational strategies, the yield of the SP1.1pts-/pSC6.090B strain is far from the theoretical maximum yields of PTS^−^ derivative strains. In 2010, Escalante et al. presented a JM101 PTS^−^ derivative strain with high glucose consumption capacity that was capable of overexpressing the *galP* and *glk* genes and that was produced from an adaptive evolution process. This strain (PB12), along with a two-plasmid expression system for *aroG^fbr^*-*tktA* and *aroB*-*aroE*, respectively, under *lac*UV5 promoters inducible by IPTG (PB12.SA22), allowed a yield of 29% (mol/mol) (Escalante et al., 2010). Further modifications allowed them to find that a *pykF* deletion could result in higher yields of total aromatic compounds, up to 50% (mol/mol), even when presenting an SA yield diminution (0.21%). In this case, the amount of flux reduced from PEP to PYR was redirected throughout the SA pathway, and without the correct amounts of enzymes, new bottlenecks appeared, causing other metabolites and intermediates to accumulate (Escalante et al., 2010). Therefore, it was clear that regulating gene expression and dosage remained a problem for more efficiently redirecting flux not only toward but also within the SA pathway. Regarding that topic, Rodriguez et al. utilized the PB12 *pykF*^−^*aroKL*^−^ strain and developed a plasmid with a constitutively strong promoter onto a synthetic operon containing the *aroB*, *tktA*, *aroG*^fbr^, *aroE*, *aroD*, and *zwf* genes (AR36) for synchronous expression of the relevant genes found in previous research. With this expression design, the AR36 derivative strain is able to redirect the carbon flow to SA even in high glucose conditions (above 100 g/L of the initial substrate concentration) without producing high acetate titers. This strain produced up to 43 g/L of SA via simple batch processes, with SA yields of 42% (mol/mol) and total SA pathway intermediate yield up to 67% of the theoretical maximum, representing the highest yield managed to be produced to date (Rodriguez et al., 2013) (Table 1; Figure 3).

Regarding the expression and regulation of key SA production genes, most of the research has been performed using plasmid expression; however, there are multiple drawbacks, ranging from structural and segregational instability to metabolic burden, of plasmid replication. Cui et al. (2014) resolved this problem by constructing a strain with an *aroG*^fbr^, *aroB*, *aroE*, and *tktA* gene cluster integrated into the chromosome and by tuning the copy number and expression by using chemically induced chromosomal evolution (CIChE) with triclosan. They also overexpressed the *ppsA* and *csrB* genes to enhance the PEP pyruvate pool. This strain rendered a 1.70 g/L SA titer, with a yield up to 0.25 (mol/mol). Finally, they studied and improved cofactor availability for SA production optimization; in this case, NADPH availability was increased because *aroE-*encoded enzymes require this specific cofactor for the DHS to SA conversion reaction. By plasmid-based or chromosomal overexpression of the NADPH availability-related genes *pntAB* or *nadK*, this cofactor pool was enhanced, which was directly correlated to the SA production capabilities of the strain. As they changed the promoters and the expression of all the chromosomally inserted genes related to SA production mentioned above, they managed to construct a strain capable of producing a yield of 0.33 (mol/mol) SA from glucose (Figure 3).

Many other examples of SA production platforms in *E. coli* have been studied in the literature, the most relevant of which are referred to in Table 1, rendering many industrially competitive strains and processes. Nevertheless, the main efforts throughout the past two decades were directed toward a particular pathway approach. As shown in Table 1, few SA production processes have been designed utilizing an overview of global regulation and manipulation, which can be obtained from omics data. Transforming this global information into global knowledge on the complexity of cell regulation would reveal the existing regulatory bottlenecks, allowing us to metabolically engineer potential strains using a systems biology approach, finally ensuring a truly rational strain design with optimized production capabilities.

## Omics Approaches for the Study of the SA Pathway in *Escherichia coli*

Classical ME approaches applied to diverse *E. coli* strains to obtain SA-overproducing derivatives have targeted key genes in the CCM and SA pathways, allowing successful reconfiguration of the biochemical network of engineered strains and resulting in the efficient redirection of carbon flow from CCM to SA production. However, the inactivation of key genes coding for enzymes involved in global regulatory processes, such as the PTS system or coding for key node enzymes, such as the PykF enzyme results in global metabolic reconfiguration, which frequently introduces significant flux imbalances. This often produces undesirable outcomes, including the accumulation of intermediates, feedback inhibition of upstream enzymes, the formation of unwanted byproducts, and the diminution of cellular fitness via the rerouting of resources toward the unnecessary or non-essential production of pathway enzymes. By understanding these newly created flux imbalances in SA-overproducing derivative strains, it is possible to boost the overall cellular physiology, product titer, productivity, and yield, taking into account a global view of cellular metabolism (Biggs et al., 2014). Combinatorial approaches allow researchers to work with this scenario by conducting global cellular searches, but the necessity for high-throughput screening is often a drawback for pathway engineering. The other approach is to augment knowledge and computational tools to properly predict designs to achieve a desired metabolic outcome (Fong, 2014). Several high-throughput approximations, such as genomic, transcriptomic, and proteomic predictions, have been applied to aromatic AAs and engineered SA-overproducing strains for the identification of non-intuitive targets other than those genes/enzymes involved in the CCM and SA pathways that might be suitable for further modification by ME.

### The identification of YdiB (*ydiB*) as a key enzyme in byproduct formation during SA synthesis

The analysis of available genome sequences using Hidden Markov Model profiles to identify all known enzymes of the SA pathway has shown that some genes have been lost in diverse microbial groups, particularly in host-associated bacteria (Zucko et al., 2010). This condition has been proposed to result in the development of undesirable metabolic traits, such as the hydroaromatic equilibration observed in *E. coli*, resulting in the synthesis of so-called missing metabolites, such as quinic acid (QA) and DHQ, by a reversion of the SA biosynthetic pathway (Knop et al., 2001; Zucko et al., 2010). The coproduction of high quantities of the byproducts DHS and QA is not a desirable trait; they significantly reduce the SA yield because QA is co-purified during the downstream process of SA purification from the culture supernatant (Knop et al., 2001; Krämer et al., 2003; Diaz Quiroz et al., 2014).

**Figure 4:**
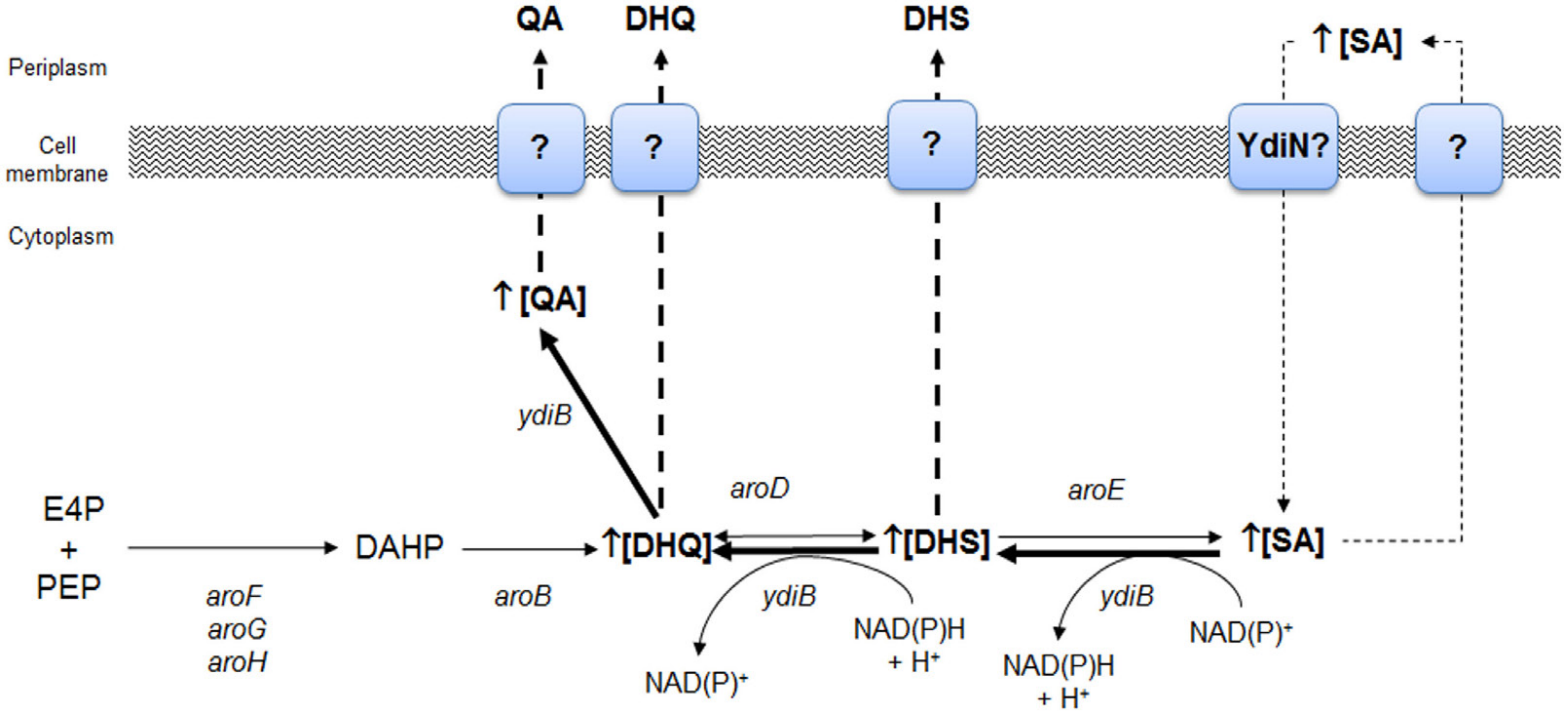
**Identification of key genes of the SA pathway involved in the biosynthesis of aromatic byproducts QA and DHS from SA as determined by global transcriptomic analysis in *E. coli* W3110.shik1**. Overexpression of *ydiB*, *aroD*, and *ydiN* genes allowed proposing that under carbon limiting growth conditions, SA is intracellularly accumulated as consequence of an inefficient export to periplasmic space or as consequence of its back transport to the cytoplasm as consequence of extracellular accumulation. YdiN, a putative transporter coded by *ydiN* was proposed to be involved in SA back import. Backflow of SA to DHS was possibly catalyzed by YdiB, whereas synthesis of DHQ from DHS was performed by AroD enzyme and finally, YdiB performed synthesis of QA from DHQ. Adapted from Johansson and Lidén (2006).

The strain W3110.shik1 (Δ*aroL*, *aroG*^fbr^, *trpE*^fbr^, and *tnaA*) engineered for SA production growing in low glucose (high phosphate) or glucose-rich (low phosphate) conditions resulted in the production of SA in cultures with mineral broth, as the single inactivation of shikimate kinase II (*aroL*) allows carbon flux to CHA through shikimate kinase I (*aroK*), resulting in the synthesis of aromatic AAs. However, under carbon-limited conditions, SA production decreased by 59%, and the byproducts DHS, DHQ, gallic acid (GA), and QA were detected in the culture supernatant with respect to phosphate limiting culture conditions (Johansson et al., 2005). Global transcriptomic analysis (GTA) of the strain W3110.shik1 in chemostatic culture conditions, comparing between glucose and phosphate limiting conditions, allowed identification of the significantly upregulated genes *ydiB* (coding for shikimate dehydrogenase/quinate dehydrogenase), *aroD*, and *ydiN*, which encodes a putative transporter, in carbon limiting conditions. The upregulation of these genes, particularly *ydiB* (10× with respect to its paralogs, *aroE*), was proposed to increase the YdiB level, which uses DHQ and SA as substrates, as this enzyme has a lower *K*_m_ for SA in the presence of NAD^+^ (Keseler et al., 2013). Additionally, the intracellular concentration of NAD^+^ is reported to be 40-fold higher than that of NADH^+^, suggesting that the dehydrogenase activity on SA to produce DHS is favored by YdiB *in vivo* (Johansson and Lidén, 2006). These results suggests that byproduct formation during SA production was associated with the reversal of the biosynthetic pathway from (1) SA + NAD(P)^+^ ↔ DHS + NAD(P)H + H^+^ and (2) DHS + NAD(P)H + H^+^ ↔ QA + NAD(P)^+^ by YdiB or (3) DHS + H_2_O ↔ DHQ by AroD (Figure 4). The presence of a large amount of intracellular SA was proposed to drive the reversal of the pathway, whereas YdiN was proposed to be the exporter of the aromatic byproducts (Johansson and Lidén, 2006).

As these results suggest an important role of YdiB in byproduct synthesis during SA production and its intracellular accumulation under glucose limiting conditions, a rational strategy to avoid byproduct synthesis was the inactivation of *ydiB* and/or the upregulation of its paralogs, *aroE*, coupled to efficient SA secretion from the cell. The upregulation of *aroE* expression (simultaneously with other key genes of the CCM and SA pathways) in PTS^−^ gluc^+^*aroK*^−^*aroL*^−^ engineered strains resulted in the highest SA titer and yield reported with low byproduct formation (Chandran et al., 2003; Rodriguez et al., 2013) (Table 1).

The replacement of *ydiB* by its paralogs, *aroE*, in a modular biosynthetic pathway design for l-tyrosine production in *E. coli* MG1655 resulted in the elimination of a bottleneck caused by the high affinity of YdiB protein for the accumulation of QA and DHS. This replacement in the modular plasmid construction P*_lac-UV5_aroE*, *aroD*, *aroB*^op^, *aroG*^fbr^, *ppsA*, *tktA* (op = optimize codon usage) resulted in the accumulation of 700 mg/L of SA, which was in turn successfully channeled to l-tyrosine (Juminaga et al., 2012). However, combinational plasmid overexpression of the *aroB*, *aroD*, *aroE*, *ydiB*, *aroK*, *aroL*, *aroA*, *aroC*, and *tyrB* genes with *ydiB* resulted in high l-tyrosine production. This result suggested that *ydiB* but not its paralog, *aroE*, is an attractive target for the overproduction of this aromatic AA because *aroE* in *E. coli* codes for a feedback-inhibited shikimate dehydrogenase, resulting in a bottleneck for l-tyrosine production (Lütke-Eversloh and Stephanopoulos, 2008).

### The impact of *pykF* inactivation on the protein levels of SA pathway enzymes

The pyruvate kinase isoenzymes Pyk I and Pyk II (coded by *pykF* and *pykA*, respectively) play key roles in CCM via Pyk activity, together with 6-phospho-fructokinase I (coded by *pkfA*) and glucokinase (*glk*), controlling carbon flux through the glycolytic pathway (Keseler et al., 2013). Pyk I and Pyk II are key allosteric enzymes that catalyze one of the two substrate-level phosphorylation steps yielding ATP and the irreversible trans-phosphorylation of PEP and ADP into PYR and ATP, maintaining a permanent flux of PYR to acetyl-CoA (Keseler et al., 2013).

Inactivation of the *pykF* gene in *E. coli* PTS^−^ derivatives (PB12 strain) engineered for SA production has resulted in the increased flux of carbon into the SA pathway (Escalante et al., 2010), increasing the DAHP concentration above 370% (and the total SA pathway aromatic yield) with respect to the *pykF*^+^ parental strain. Further applications of ME strategies in the PB12 strain *pykF*^−^ resulted in the derivative strain AR36, which produces up to 40 g/L SA with a yield of 0.42 mol SA/mol glc (Table 1) (Rodriguez et al., 2013), demonstrating that the inactivation of *pykF* in a PTS^−^ derivative strain significantly improves PEP flux toward SA synthesis.

Global proteomic analysis in a *pykF*^−^ derivative of *E. coli* (BW25113) compared with its *pykF*^+^ parental strain revealed the differential overexpression of 24 proteins, including enzymes from the SA pathway and aromatic AAs. The upregulation of key SA pathway enzymes, including the DAHPS AroG isoenzyme (2.66 times more abundant with respect to the *pykF*^+^ strain), which is involved in the synthesis of DAHP, the first intermediate of the SA pathway, and the AroB enzyme (DHQ synthase, 4.72 times more abundant with respect to the *pykF*^+^ strain) (Kedar et al., 2007). These results support the positive impact of *pykF*^−^ inactivation not only on increased PEP availability but also on increased carbon flux toward the SA pathway.

### The identification of other possible key catabolic and biosynthetic genes involved in SA production

Batch fermentation cultures of the *E. coli* PB12.SA22-derivative strain for SA production (PTS^−^ Glc^+^*aroK*^−^, *aroL*^−^*aroG*^fbr^, *tktA*, *aroB*, and *aroD*; Table 1) using complex production media containing 25 g/L glucose and 15 g/L yeast extract (YE) showed two characteristic growth stages: a fast growth phase associated with low glucose consumption during the first 8–10 h of cultivation and low SA production, and a second slow growth stage with high glucose consumption until this carbon source was completely consumed (25 h of cultivation). Interestingly, SA production continues during the STA phase after glucose, used as a carbon source, was completely consumed, until the end of fermentation (50 h) (Escalante et al., 2010). This behavior suggested that during the EXP growth phase, this strain preferentially consumed some YE components to support growth, whereas glucose was used to produce SA and other pathway intermediates, suggesting the existence of regulatory and physiological differences between EXP and STA phases (Cortés-Tolalpa et al., 2014).

**Figure 5:**
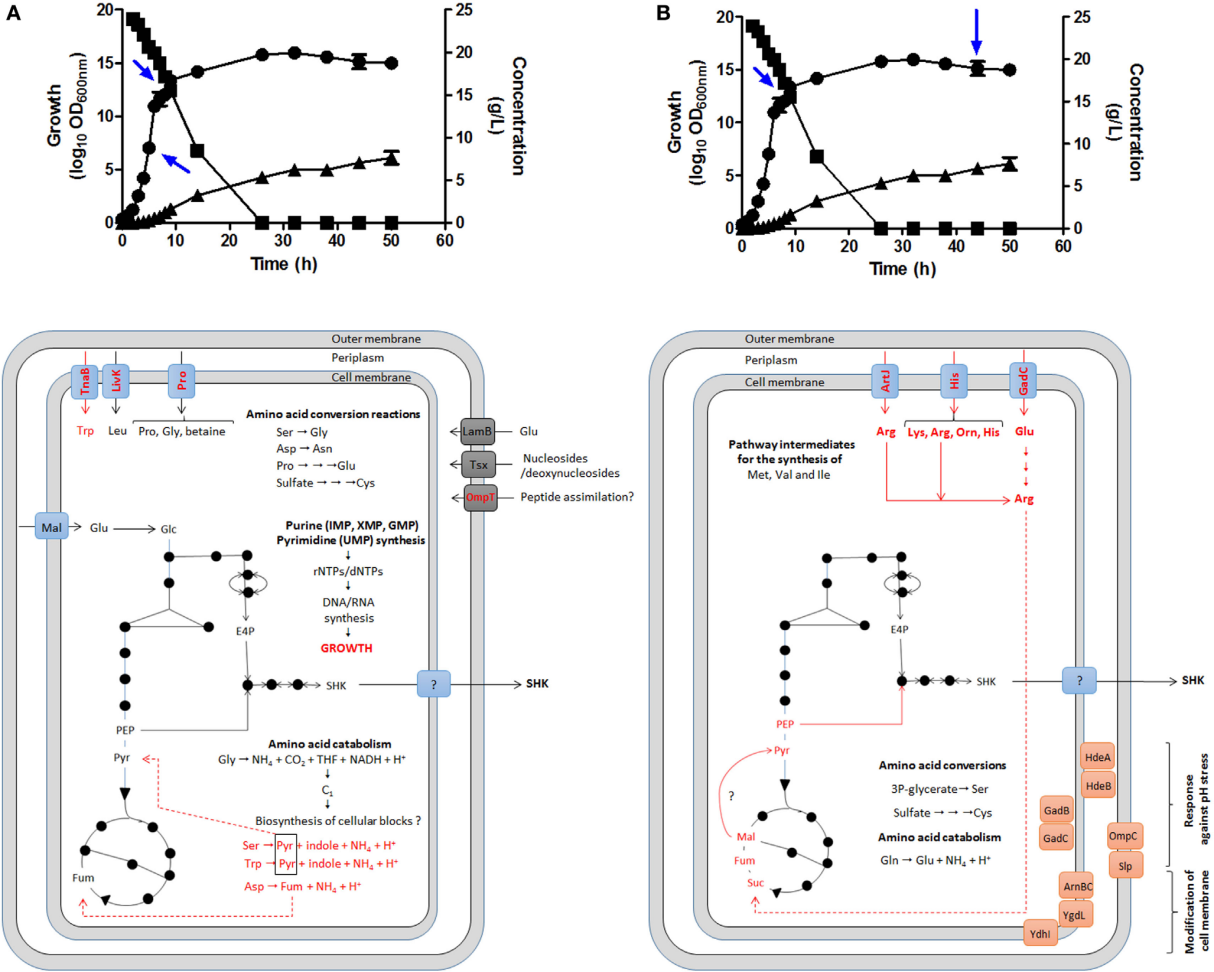
**Identification of possible key genes involved in carbon supply for SA synthesis as determined by global transcriptomic analysis in *E. coli* PB12.SA22 in batch culture using complex fermentation broth**. Global transcriptomic analysis (GTA) showed no changes in expression profile in comparisons between EXP/STA1 **(A)** and STA1/STA2 **(B)** stages of those genes coding for enzymes of CCM and SA pathways but differential overexpression of diverse genes involved the transport, catabolism and interconversion of amino acids was observed (in red color) [**(A,B)**, lower panels]. During STA1/STA2 comparison, genes coding for l-arginine, l-lysine, l-glutamic acid, and l-ornithine transporters were upregulated. These amino acids are probably converted to succinate fueling carbon to TCA. Additionally diverse genes coding for stress response proteins to pH and osmotic pressure were overexpressed. Blue arrows in upper panels showed samples from fermentor culture analyzed for GTA. Growth (•), glucose consumption (▪), and SA production (▴). Adapted from Escalante et al. (2010), Keseler et al. (2013), and Cortés-Tolalpa et al. (2014).

GTA was performed to corroborate this hypothesis during SA production in batch fermentation cultures using complex fermentation broth (Chandran et al., 2003; Escalante et al., 2010; Rodriguez et al., 2013) by comparing global expression profiling between the mid-exponential growth phase (EXP, 5 h of cultivation), the early stationary phase (STA1, 9 h) and the late STA phase (44 h); EXP/STA1, EXP/STA2, and STA1/STA2 comparisons were conducted (Cortés-Tolalpa et al., 2014) (Figure 5).

The relevant results showed EXP growth in the derivative strain PB12.SA22 during the first 9 h of cultivation. When the l-tryptophan provided by YE available in the supernatant was completely consumed (6 h), the strain entered the low-growth phase (even in the presence of glucose) until 26 h of cultivation, when glucose was completely consumed; this was associated with low SA production. Interestingly, during the stationary stage, SA production continued until the end of fermentation (50 h), achieving the highest accumulation (7.63 g/L of SA) in the absence of glucose (Figures 5A,B, upper panel).

GTA comparisons among EXP/STA1, EXP/STA2, and STA1/STA2 showed no significant differences in the regulation of genes from the CCM and SA pathways, but for the EXP/STA1 comparison, the upregulation of genes coding for sugar transport, AA catabolism and biosynthesis, and nucleotide/nucleoside salvage was observed (Figure 5A). Interestingly, in the STA2 phase, the highest SA production was observed in the absence of glucose in supernatant, associated with the upregulation of genes encoding transporters for the AAs l-lysine, l-arginine, l-histidine, l-ornithine, and l-glutamic acid and enzymes involved in the synthesis, interconversion, and catabolism of l-arginine. As all of these AAs are provided by YE, this result suggests that this AA could play a key role in fueling carbon to SA synthesis, and likely also in l-arginine conversion to the TCA intermediate succinate through the super-pathway of l-arginine and l-ornithine degradation (Keseler et al., 2013) (Figure 5B). These results indicate the origin of carbon required for the highest SA production during the STA phase after glucose was completely consumed. Additionally, the upregulation of genes involved in the pH stress response and inner and outer membrane modifications suggests a cellular response to environmental conditions imposed on the cell at the end of fermentation (44 h) (Cortés-Tolalpa et al., 2014).

The upregulation of genes coding for the biosynthesis and interconversion pathways of almost all AAs was also observed by GTA in cultures under C-limiting condition of the derivative strain W3110.shik1 grown in minimal broth. These changes were postulated to correlate to aromatic AA starvation with these culture conditions, although this strain maintained functional shikimate kinase I (*aroK*), allowing the accumulation of SA but maintaining carbon flux toward CHA and aromatic AAs (Johansson and Lidén, 2006).

As demonstrated by GTA in the SA-producing strain PB12.SA22 during batch culture fermentations in complex media containing YE, several metabolic constraints limit the growth capabilities of this strain, stopping growing even in the presence of glucose. The highest SA production observed in the late stationary stage in the absence of glucose was probably supported by the non-aromatic AA content of YE. This evidence supports valuable information to further optimize culture strategies, as YE feeding increased the SA titer and yield in engineered strains.

## Omics Data Integration into Metabolic Modeling: Moving toward Data Integration for Rational Strain Improvement

Although ME is capable of reconfiguring a biochemical network to redirect the substrate conversion into valuable compounds by manipulating the microorganism genetic code, its classical rational approach often introduces significant new flux imbalances. This has often caused undesirable outcomes due to the accumulation of intermediates, feedback inhibition of upstream enzymes, and the formation of unwanted byproducts of cellular fitness diminution via the rerouting of resources toward the unnecessary or non-essential production of pathway enzymes (Biggs et al., 2014). By understanding these newly created flux imbalances on mutant strains, it is possible to boost overall cellular health and the product titer, productivity, and yield, taking into account a holistic view of cellular metabolism (Biggs et al., 2014). Since the development of the omics, there has been an increased interest to understand the behavior of complete biological systems. Omics renders biological data from all levels of metabolism going all the way from genome to metabolome, these data combined give us the possibility to study the whole organism instead of single components. To achieve this, mathematical models play the important role of converting omics data into organismal information and knowledge (Åkesson et al., 2004; Fong, 2014). There are several frameworks and approaches for the mathematical modeling of metabolism developed to collect high-throughput data to understand as well as to predict phenotypic function. Computational applications have been developed using models as quantitative mathematical representations of biological systems and or their components to a suitable level of simplification (Jouhten, 2012). These computational tools can be used to identify new biological pathways in the host microorganism for the selection and improvement of important genotypic characteristics to improve the production of the desired compound (Long et al., 2015). In this section, we discuss some mathematical models and computational tools that can be used in ME to utilize all high-throughput omics data and render new insights into flux distributions, regulation constraints, and modification targets to optimize the production of desired metabolites.

To understand the challenges and virtues of mathematical modeling, we must observe that biological systems are complex in nature, involving the transport of information through many layers, including the genome, transcriptome, proteome, and metabolome; therefore, regulatory steps between the interactions of these layers finally render the complex outcome of the phenotypic behavior (Cloots and Marchal, 2011; Fong, 2014). Therefore, mathematical models have been used to evolve and clarify the complex network interactions and system characteristics to reveal the underlying mechanisms. Despite this high degree of complexity, with all the recent advances and data sets available, mathematical modeling promises to generate experimentally testable hypotheses, predictions, and new insights into systems biology to better understand cell behavior (Stelling, 2004).

The first step in mathematical modeling is reconstructing the metabolic network. With the advent of the genomic era since approximately 1999, reconstruction can be achieved on a genome-wide scale for many organisms and has been used to expand the knowledge on metabolic networks and to identify new or non-intuitive metabolic reactions to be engineered for further strain improvements (Åkesson et al., 2004; Kim et al., 2012). Genome-scale models are assembled and manually curated from the annotated genome, and biochemical information is used to render a representation of the metabolic network on which mathematical representations will set a matrix of equations to model its behavior. The reconstruction of a genomic metabolic network starts through the examination and identification of the coding regions or open reading frames on the sequence. After analysis with established algorithms and biochemical and physiological databases (EcoCyc, MPW, and KEGG WIT), sequences can be converted into feasible reactions, and a metabolic network can be reconstructed from genomic information (Covert et al., 2001). This reconstructed network, based on genomic data, is now the backbone of an *in silico* organism. Many organisms have been completely sequenced and have simultaneously been extensively biochemically studied, which in turn can make the reconstructed metabolic network more complete (Covert et al., 2001). In recent years, ~40% of all eukaryotic models and 30% of the total prokaryotic models have been published, advancing from highly characterized organisms (*E. coli* and *Saccharomyces cerevisiae*) to less characterized species with more complex biological systems that have special characteristics for specific applications (Kim et al., 2012). When a network is described with sufficient detail, some qualitative predictions can be made, and with the inclusion of stoichiometric, thermodynamic, and kinetic data, the reconstructed metabolic map of an organism can be used to generate quantitative predictions regarding phenotype via the construction of mathematical models (Covert et al., 2001). For example, individual genes have been deleted from *in silico* models, and correlations between the model and experimental data for the consequences of each deletion have been found to be 60% accurate for *Helicobacter pylori* and 86% accurate for *E. coli* (Price et al., 2003). Nevertheless, the challenges for the construction of these *in silico* models include obtaining high-throughput data to reconstruct more complete models, which can be sorted out by using omics data and combinatorial experimentation, and constructing mathematical approaches to model and render specific solutions for the highly complex systems of biological networks. Because genome-scale metabolic networks comprise hundreds to thousands of reactions, a large number of parameters are required to mathematically describe networks, which, therefore, requires the development of informatic intensive modeling approaches to describe its complexity and to make useful predictions regarding phenotypic behavior for strain design (Price et al., 2003).

The most used approaches are those arising from stoichiometric modeling, which uses mass balances over the metabolic network and assumes a pseudo-steady-state condition to determine intracellular metabolic fluxes, along with additional experimental data to solve the underdetermined linear equation system (Åkesson et al., 2004). Stoichiometric modeling creates a matrix (*S*) for the metabolites and metabolic reactions, in which each element indicates a stoichiometric coefficient, along with a vector that contains all of the unknown reaction rates (*v*); under the steady state assumption, flux distribution will be represented by *S.v* = 0 (Jouhten, 2012; Kim et al., 2012). As expected, this equation system will have many solutions, or more precisely, it will render a convex solution space, and because genome-scale metabolic models include all possible metabolic reactions whether or not they are expressed, meaningful solutions must be narrowed down to render a viable solution (Kim et al., 2012). The main problem is that due to the high number of equations and parameters, these systems are always underdetermined; thus, the use of thermodynamic, metabolic, kinetic, and all other experimental data available is required to impose constraints, to reveal a plausible solution, and therefore to conduct quantitative analysis and make predictions regarding cell behavior (Fong, 2014).

To accomplish this desirable outcome, mathematical modeling researchers have developed many approaches to render the complexity, including the use of interaction-based, constraint-based, and mechanism-based methodologies for calculations. Interaction-based approaches isolate autonomous units performing distinct functions in cellular systems, accounting for modularity, which simplifies networks and systems to perform a topological analysis to reveal the principles of cellular organization. Constraint-based approaches account for the physicochemical invariance of networks in addition to network topology. This approach along with stoichiometric modeling, is capable of confining the numerous steady-state flux distributions the metabolic reconstruction network can have (convex space of solutions) into a smaller group, which complies with the constraints indicated by the knowledge regarding the system (a set of feasible states). Even so, this approach accounts only for the steady state, and therefore produces static models; thus, the final phenotypic behavior in changing intracellular or extracellular environments is difficult to address (Stelling, 2004). Mechanism-based approaches use kinetic parameters along with stoichiometric parameters to render the dynamic behavior of cells; thus, such approaches can formulate precise flux distributions and explore the regulation over time. The main problem with this approach is that the knowledge on mechanisms and associated parameters (kinetic reaction parameters) has, thus, far been limited, as so much effort and so many resources must be used to accomplish this type of models (Stelling, 2004; Jouhten, 2012; Long et al., 2015).

Constraint-based approaches are the most used ones to date because of their capability to render flux distribution modeling even with a relatively small amount of information. These approaches state the constraints under which the reconstructed network operates based on stoichiometry and thermodynamics, including directionality and biochemical loops (Price et al., 2003). Such constraints can be imposed by linear optimization; for example, standard flux-base analysis (FBA) uses growth optimization, selecting only the flux solutions, that in turn, produce the maximum growth rate for network topology (Åkesson et al., 2004). Newer flux solution reduction methods have been developed to study the solution space, accounting for the optimization of not only growth but also many other linear and non-linear objective functions, such as the maximum biomass, maximum ATP, minimum overall intracellular flux, maximum ATP yield per flux unit, maximum biomass yield per flux unit, maximum substrate consumption, minimum number of reaction steps, minimum redox potential, and minimum flux production between others (Price et al., 2003; Schuetz et al., 2007). These optimization principles, along with other constraints arising from specific conditions being either biotic (e.g., the inactivation, subexpression, or overexpression of specific target genes) or abiotic (e.g., aerobic culture, anaerobic culture, nitrogen limitation, carbon limitation, available substrates), will help not only to render the most feasible flux distribution solution but also to study the consequences of changing the genetic cellular output or fermentation parameters for a specific objective. This information is of great use for ME because it renders the ability through different modeling frameworks to study and predict the effects of knocking out genes, tuning the expression of target genes involved in specific reactions, network robustness, the endpoint of adaptive evolution, the identification and characterization of regulation, and heterologous reactions and *de novo* reactions on strain design. There are many reviews that discuss and compare multiple modeling frameworks, such as OptGene, OptStrain, CosMos, OptFocrce, FaceCon, and FOCAL, for the constraint-based analysis of genome-wide metabolic networks (Price et al., 2003; Schuetz et al., 2007; Krull and Wittmann, 2010; Cloots and Marchal, 2011; Jouhten, 2012; Fong, 2014; King et al., 2015; Long et al., 2015). In this review, we will focus only on one or two framework examples given the scope of this work.

The first strain design method involving knockouts is OptKnock, a bi-level optimization framework used to identify optimal reaction deletion strategies, coupling cellular growth, and target metabolite production. OptKnock identifies deletions with the highest chemical production within the solution space obtained by the maximum growth rate constraint (Long et al., 2015). Pharkya et al. (2003) used this framework to explore the overproduction of amino acids; specifically for AA, they addressed the channeling of flux from PEP to AA by removing the *ppc* gene, which could lead to the redirection of carbon flux to the formation of CHA via the accompanying deletions of pyruvate oxidase, pyruvate dehydrogenase, and pyruvate lyase reactions. The deletion of *ppc* by itself fails to redirect PEP to AA; the ability to detect its contribution though the co-inactivation of other reactions is a very useful tool of ME because the classical experimental deletion of this gene would have produced negative results for pathway optimization. In other words, *in silico* modeling enables researchers to avoid designs toward a local maxima or minima when trying to identify the modifications required to achieve a global maxima for their specific purposes.

FBA with grouping reaction constraints (FBAwGR) was developed to improve the accuracy of metabolic simulation by incorporating the grouping of reaction constraints of functionally and physically related reactions in the model. This framework allows the consideration of genomic context and flux-converging analyses. Genomic context accounts for conserved neighborhoods, gene fusion, and co-occurrences of genes to organize fluxes that are likely to be on or off together. Flux-converging analyses then restrict the carbon flux solution space to the number of metabolites participating in reactions and converging patterns from a specific carbon source. This framework has been used to predict changes in flux patterns caused by several genetic modifications, such as *pykF*, *zwf*, *ppc*, and *sucA* deletions in *E. coli*, showing good agreements with experimentally obtained fluxes (Kim et al., 2012).

Regarding the scope of this review for SA production in *E. coli*, we have found few studies in the literature that account for metabolic modeling. Nevertheless, the notable work by Chen et al. (2011), described FBA constraint analysis by stoichiometry and mass balance, assuming no growth and optimizing SA as the objective function to design modifications for the production of intermediate metabolites of the aromatic pathway. The model identified several key reaction steps for overexpression, similarly to those previously reported for AA optimization (overexpression of the *aroF*, *tktA*, *ppsA*, and *glf* genes, as well as deletions of the *ldhA* and *ackA* genes) by avoiding carbon waste through lactate and acetate fluxes. Finally, with all of the modifications made, their model identified the *zwf* gene as the critical node for redirection of the carbon flux into the AA pathway; its deletion led to an optimized accumulation of QA, GA, and SA, accounting for a 47% molar conversion of glucose (Chen et al., 2014).

Regarding other SA related work, Rizk and Liao (2009), managed to use EM to model, study, and predict DAHP production in *E. coli* toward aromatic production. Ensemble modeling (EM) is a mechanism-based modeling approach that decomposes metabolic reactions into elementary reaction steps, incorporating all available phenotypic observations for the wild type and mutant strains, integrating this information into the mathematical approach to identify the kinetic variables of each elementary reaction step (Rizk and Liao, 2009; Khodayari et al., 2014). Rizk and Liao (2009), using different flux bounds on the pathway split ratio between glycolysis and the PPP. Then, by using data from literature for overexpression of the *tktA*, *talA*, and *pps* genes, they were able to screen the solution space models compared with the phenotypic behavior, selecting the ones that properly described the experimental data (from a 1500 solution space to 7, 171, and 195 solution spaces, according to glycolysis:PPP ratios of 25:75, 75:25, and 95:5, respectively). This subset of flux solutions revealed that TktA is the first controlling rate step and that PPS, only with simultaneous overexpression of TktA can augment DAHP production; these findings are in accordance with the phenotypic observations in the literature. Based on these results, they conclude that the flux distributions found could be reverse engineered to enhance aromatic production in *E. coli* (Rizk and Liao, 2009).

Notably, despite the existence of many genome-scale metabolic models and various mathematical approaches, many of the fluxes remain undetermined, as many solutions remain plausible. Thus, more information is needed to ensure the modeling quality by the validation and incorporation of *in vivo* experimental data. These experimental data can be acquired from transcriptomic, proteomic, or fluxomic data. Strategies incorporating these extensive experimental data have been developed to enhance the quality and the accuracy of metabolic models (Kim et al., 2012). Fluxomic data in its core provide us with the most important information as fluxes are the modeling outcome, but experimental procedures can only be used for relatively smaller networks and in specific conditions. Nevertheless, these data are of utmost importance and are commonly used to validate model solutions to flux distributions. ME models have been used to integrate protein expression data to reconstruct and add constraints to genome-level metabolic models, relating kinetic equations into catalytic constrains to approximate stoichiometric relationships between enzyme abundance and catalyzed fluxes (O’Brien and Palsson, 2015). This integration of proteomic data adds thermodynamic and allocation constraints that help in the identification of a consistent flux state, allowing an explanation of aspects of cell behavior and relationships that have remained elusive, such as the interaction of ribosomes with metabolism, carbon limited to carbon excess metabolic shifts, substrate uptake regulation, membrane protein relationships, and other protein spatial constraints that can utterly dominate and/or change metabolic responses (O’Brien and Palsson, 2015). Transcriptomic data have been used to exploit the regulatory information in the expression data to provide additional constraints for the metabolic fluxes in the model by analyzing if or when gene expression correlates with a given metabolic flux (Åkesson et al., 2004; Kern et al., 2006). Computational protocols have been developed for this type of data integration, such as mixed integer linear programing (MILP), which seeks to maximize the agreement between experimental data and computational fluxes by limiting the presentation of entities with the capability to carry flux; meanwhile, the flux of absent entities would be 0 (Fong, 2014). Åkesson et al. (2004) used gene expression microarray data from chemostat and batch cultures of *S. cerevisiae* to create Boolean variables for all of the reactions encompassed on a genome-scale metabolic model to ascertain the absent/present fluxes using analysis software. These new constraints allowed the computation of metabolic flux distributions to enhance the metabolic behavior in batch cultures, along with the quantitative prediction of exchange fluxes as well as the qualitative estimation of changes in intracellular fluxes compared with the model without transcription constraints, as verified by experimental measurements of flux (Åkesson et al., 2004).

Many methods have been developed to introduce transcriptomic regulation into modeling predictions, such as probabilistic regulation of metabolism (PROM), which calculates the probability that a metabolic target gene will be expressed relative to the activity of its regulating transcription factor, metabolic adjustment by differential expression (MADE), which creates a sequence of binary expression states so that when the gene expression changes from one condition to another, the flux reaction will change in accordance with its value, and gene inactivity moderated by metabolism and expression (GIMME), which is a context metabolic model that predicts the subsets of reactions used under a particular condition using gene expression data and which identifies a flux distribution to optimize a given biological objective, such as growth and/or ATP production, along with FBA (Kim et al., 2012). Finally, a method called E-Flux can map continuous gene expression into flux bound constraints according to gene–protein-reaction (GPR) associations, limiting the upper and lower bounds on fluxes so that genes expressed at higher levels will result in higher flux values (Kim et al., 2012). This and other methods have been reviewed and compared by Machado and Herrgård (2014), who concluded that the prediction of flux levels from gene expression remains far from solved because the predictions obtained by simple FBA with growth maximization and parsimony criteria were as good or even better that those obtained using the incorporation of transcriptomic data. Nevertheless, they acknowledge that some methods evaluated give reasonable predictions under certain conditions that there is no universal method that performs well under all scenarios and that the transcriptome should provide some guidelines for the correct phenotype determination within the space of solutions resulting from the large number of degrees of freedom in metabolic networks, recommending that users should perform a careful evaluation of the meaningfulness of the results for their particular applications (Machado and Herrgård, 2014).

There are many successful mathematical modeling approaches to produce good and accurate predictions of phenotypic behavior in the literature; all of these methods help us to understand and simplify metabolic regulation and systems to comprehend and find new or non-intuitive targets for ME. Even so, there is still much work to be conducted to understand and construct better models of metabolic networks. There are many challenges because cell behavior is a complex system that, therefore, has complex outcomes and regulation. These challenges range from network reconstruction, mathematical treatments, and true flux distribution determination to the integration of all systems data (omics) to achieve regulation and phenotypic predictions. Nevertheless, the effort put into understanding this matter has produced and will continue to produce new insights for strain design and ME. Explaining all the considerations, challenges and achievements in this field is not within the scope of this review as many reviews have been published on these matters (Liu et al., 2004; Patil et al., 2004; Stelling, 2004; Schuetz et al., 2007; Kim et al., 2012; Machado and Herrgård, 2014; Saha et al., 2014; Long et al., 2015; O’Brien and Palsson, 2015). Rather, this review is aimed to provide the reader with interesting findings and perspective on how the mathematical modeling of biological systems can be and is useful for ME, especially regarding SA and AA production, for which these methods can be of relevance to exploit the maximum production capability of *E. coli* that remains unachieved.

## Summary and Perspectives

SA is a key intermediate of the common aromatic pathway with diverse applications in the synthesis of valuable pharmaceutical compounds, but major interest relies on SA as the precursor for the chemical synthesis of OSF, the neuraminidase inhibitor of diverse influenza viruses, including pandemic strains. Diverse efforts have been made to produce high titers and yields of SA in metabolically engineered strains of *E. coli* with successful genetic modifications, including the following: (1) interruption of the SA pathway by the inactivation of shikimate kinase coding genes (*aroK* and *aroL*), which results in the high accumulation of SA; (2) increasing the intracellular availability of the CCM intermediate PEP by inactivation of the PTS system and replacing this glucose translocation system by other housekeeping or heterologous glucose transporters and by inactivation of the *pykF* gene; and (3) the overexpression of diverse key genes of the CCM and SA pathways, such as *zwf*, *tktA*, *aroB*, *aroD*, and *aroE*, under the control of constitutively expressed or inducible promoters in plasmid-cloned operons or chromosome-integrated copies. These engineered strains have been cultured in batch or fed-batch culture conditions using a complex fermentation media including glucose and YE, resulting in the highest titer and yield of SA reported (Chandran et al., 2003; Rodriguez et al., 2013).

The above-described genetic changes impose global nutritional, regulatory, and metabolic constraints on the resultant engineered strains, which must be explored to determine their relevance on SA production. GTA of the SA-producing strain W3110.shik1 provided evidence supporting the roles of *ydiB-*, *aroD-*, and *ydiN-*encoded proteins in byproduct formation during SA production under glucose limiting conditions (Johansson and Lidén, 2006). Recent ME strategies applied for l-tyrosine (Juminaga et al., 2012) and SA production (Rodriguez et al., 2013) demonstrated the relevance of *ydiB* inactivation and *aroD* overexpression to avoid byproduct formation and to improve carbon flux toward the desired aromatic products.

Interruption of the SA pathway by inactivation of the *aroK* and *aroL* genes imposes an auxotrophic requirement for aromatic AAs and probably other metabolites derived from CHA on the cell; these effects were successfully reversed by the addition of YE to the fermentation media.

As the chemical complexity of YE or peptone significantly interferes in the study of carbon flux through the CCM and SA pathway metabolic networks, no studies to date have been reported on the application of metabolic models to identify possible targets for the application of further ME strategies focused on the improvement of SA production in fermentation culture using complex production media (Chandran et al., 2003; Escalante et al., 2010; Chen et al., 2012; Rodriguez et al., 2013; Cui et al., 2014). The application of omics, such as GTA, in SA-producing conditions, including YE, as reported for the strain P12.SA22, provides valuable information on the role of diverse transporter systems and other pathways involved in carbon supply from YE to SA synthesis (Cortés-Tolalpa et al., 2014). These results highlight the relevance of information retrieved from the application of omics, such as GTA, or proteomic approaches in successful aromatic compound-producing strains to obtain data for mathematical modeling of metabolism.

Further application of synthetic biology strategies based on modular combinational design including key genes from the CCM and SA pathways in operons and optimized codon usage, and the construction of continuous genetic modules regulated by the same promoter but coupled to an efficient translational level by the selection of efficient ribosome binding sites (RBS) from tailored-made RBS libraries are promising strategies for the subsequent optimization of SA-producing strains. These synthetic strategies have been applied for the efficient production of l-tyrosine in *E. coli* (Juminaga et al., 2012) and for the successful production of SA in *Corynebacterium glutamicm* (Zhang et al., 2015), respectively. Great advances in SA production in *E. coli* have been made over the past decades. However, more and new developments must be made, taking into account the vast, recently acquired data from omics technology. These data, along with their integration with ME technology and experience, can lead to more global insight into cell physiology, allowing new engineering techniques from a systems ME perspective to be identified and developed.

## Author Contributions

All authors participated equally in the preparation of this contribution. All authors have read and approved the final manuscript.

## Conflict of Interest Statement

The authors declare that the research was conducted in the absence of any commercial or financial relationships that could be construed as a potential conflict of interest.
